# Solvent-Cast PCL/Hemp Biocomposites: Screening of the Effects of Matrix Grade, Filler Content and Particle Size

**DOI:** 10.3390/ma19204377

**Published:** 2026-10-18

**Authors:** Gaia Francesca Lupia, Tommaso Pini, Paola Francesca Colella, Marco Valente

**Affiliations:** 1 Department of Chemical Engineering, Materials, Environment, Sapienza University of Rome, 00184 Rome, Italy; gaiafrancesca.lupia@uniroma1.it (G.F.L.); marco.valente@uniroma1.it (M.V.); 2 INSTM Reference Laboratory for Engineering of Surface Treatments, UdR Rome, Sapienza University of Rome, 00184 Rome, Italy

**Keywords:** PCL, hemp, lignocellulosic filler, biocomposites, solvent casting, particle size effect, defect-controlled behavior

## Abstract

Biodegradable poly(ε-caprolactone) (PCL)/hemp biocomposites were prepared by solvent casting in acetic acid to screen the effects of matrix grade, filler content, and particle size on tensile behavior. Two PCL grades (Capa^®^ 6500 and 6800) were combined with milled hemp sieved at four sizes (0.08 to 1.0 mm) and two contents (20 and 30 wt%), giving 13 formulations (86 specimens), analyzed statistically and with a combined Pukanszky–Weibull model. Particle size produced a plateau: the 0.08 and 0.25 mm fractions were equivalent (*p_adj_* > 0.25), with a marked drop at 0.5 mm. Raising the content to 30 wt% lowered the strength 2.5 to 4 times (*p* < 0.001), showing that hemp acts mainly as a filler. For PCL 6800 at 20 wt%, the modulus at the two finest sizes exceeded the neat value, a mild reinforcement described by a Halpin–Tsai term. A low Weibull modulus (*m_W_* ≈ 3) indicated defect-controlled behavior, and electron microscopy showed matrix voids, more pronounced for PCL 6800, and interfacial gaps. Thermal analyses indicated limited matrix degradation during dissolution and unchanged thermal stability. PCL 6800 with 20 wt% hemp at 0.25 mm performed best. This feasibility screening supports formulation selection for a planned melt processing phase.

## 1. Introduction

The environmental burden of fossil-based plastics has motivated a sustained effort toward more sustainable polymeric materials, increasingly framed within circular economy principles in which renewable sourcing, reduced carbon footprint, biodegradability and end-of-life valorization are considered together [1,2,3]. Bio-based origin and biodegradability are independent properties: a material can be bio-based without being biodegradable, and biodegradable while being derived from petrochemical sources, with compostability representing a specific case of biodegradation under controlled conditions [4]. Material selection should therefore account for performance and processability, not only for environmental considerations, and the actual benefit of a biodegradable system depends on its formulation, manufacturing route, and end-of-life scenario [3,4].

Among biodegradable polymers, PCL has attracted particular interest because of its low melting temperature, good processability and compatibility with natural reinforcements [5,6]. PCL is a semicrystalline aliphatic polyester with a glass transition temperature of approximately −60 °C and a melting temperature in the range of 58 to 64 °C [6], which make it easy to process and well suited to be the matrix of composites containing lignocellulosic phases, since the low processing temperature limits thermal degradation of the reinforcement. On the other hand, PCL exhibits an intrinsically ductile behavior and a relatively low modulus, which can limit its use where stiffness and dimensional stability are required [6,7]. For this reason, reinforcing or filler phases are frequently introduced. The incorporation of nanostructured fillers, such as layered silicates, has been reported to improve the mechanical and thermal properties of PCL through enhanced interfacial interactions [8,9], and comparable effects have been described for bio-nanocomposites in packaging applications [10].

Among lignocellulosic reinforcements, hemp (*Cannabis sativa* L.) is attractive because of its low density, favorable specific properties, wide availability and the possibility of valorizing processing by-products [11,12,13]. Hemp cultivation can require comparatively limited water and agrochemical input, although the actual footprint is strongly cultivar- and region-dependent [14]. When the reinforcement moves from the nanoscale to milled microscale particles, the composite behavior becomes more complex. Interfacial incompatibility between the hydrophilic lignocellulosic phase and the hydrophobic matrix is a known limitation and frequently governs failure more strongly than the intrinsic filler properties [15,16,17]. Hemp has been combined with PCL mostly in fiber form and by melt processing: Dhakal et al. reported that hemp fibers in PCL (Capa^®^ 6800) increased the modulus by up to about 186% depending on fiber aspect ratio, while the tensile strength was essentially unchanged, a behavior that can be attributed to the weak fiber/matrix interface [18]. Similar filler-dominated responses have been observed for particulate lignocellulosic fillers such as bamboo powder in PCL, where the modulus increased but the strength decreased [19] and for other natural fiber/PCL systems [17,20,21,22].

Particle size and filler content control filler dispersion, defect formation and stress concentration in particulate-filled polymers [23]. Coarse particles can act as flaws, whereas smaller particles tend to improve stress distribution and reduce defect severity; however, very fine particles may favor agglomeration, so a monotonic trend is not necessarily expected [23,24]. Increasing the filler content, on one hand, raises stiffness but on the other hand reduces matrix continuity and promotes a defect sensitive behavior [19]. These aspects are amplified in solution-based processes such as solvent casting, where the final microstructure depends on solvent evaporation kinetics and chain rearrangement [25,26] and where residual solvent and processing defects can lower the apparent properties relative to bulk material [27,28]. For rigid particles with poor adhesion, the modulus increases with the filler content and depends only weakly on particle size at the micrometer scale, whereas the strength decreases as particle size and content increase [23]; comparable trends were reported in PCL for bamboo powder [19] and for other agricultural wastes [29].

Hemp has been combined with PCL mainly in fiber form and by melt processing [18,20], or as long stalk fragments in porous panels for sound absorption [13]. Particulate lignocellulosic fillers were studied in PCL for other sources, such as bamboo powder [19] and agricultural wastes [29], and hemp hurd particles were investigated in PLA, also in relation to particle size and 3D printing [30]. To the authors’ knowledge, milled hemp processing residues with measured size distributions have not been investigated as fillers in dense PCL composites. In particular, the coupled role of PCL grade, hemp particle size, and filler content on the structure–property relationships and a quantitative description of the filler and defect contributions have not been explored yet. The present study is conceived as a preliminary, feasibility-oriented step toward the manufacturing of these biocomposites by melt processing, specifically injection molding and fused deposition modeling (FDM). Solvent casting is used here as a rapid, low-material-consumption screening method to establish the relationships between matrix grade, filler content, filler granulometry and mechanical response before committing to the more resource-intensive melt processing trials. The two intended routes impose different, and partly opposing, constraints on the usable particle size: FDM is limited by the filament and, above all, the nozzle diameter, so that only sufficiently fine powders can be processed without clogging [31], whereas injection molding tolerates coarser fractions [32]. Investigating the full range from 0.08 to 1.0 mm therefore brackets the particle size windows of both processes. The intended application, packaging of cosmetic products, adds further consideration: although finer particles are expected to favor the mechanical properties, the appearance and surface texture imparted by the natural filler are also relevant to the final product, so that the most suitable particle size is process- and application-dependent rather than defined by mechanical performance alone.

In the present work, biodegradable PCL/hemp biocomposites were produced by solvent casting in acetic acid, using two PCL grades and four hemp particle sizes at two filler contents. The materials were characterized by tensile testing, laser diffraction granulometry, scanning electron microscopy, and, for the neat matrices, differential scanning calorimetry and thermogravimetric analysis, and the tensile dataset was analyzed statistically and described with a combined filler/defect model. The aim was to identify the structure–property relationships associated with filler granulometry and matrix molecular weight, and to provide a quantitative basis for formulation and for a future transition toward scalable melt processing routes.

## 2. Materials and Methods

### 2.1. Materials

Two commercial grades of PCL, Capa^®^ 6500 and Capa^®^ 6800 (Ingevity, North Charleston, SC, USA), were used as matrices. Both are semicrystalline biodegradable polyesters with a density of approximately 1.1 g/cm^3^ and a melting temperature in the range of 58 to 62 °C. The grades differ in number-average molecular weight, approximately 50,000 g/mol for Capa^®^ 6500 and 80,000 g/mol for Capa^®^ 6800. The filler was a hemp (*Cannabis sativa* L.) industrial processing residue supplied by Canapack (Modena, Italy) as irregular woody fragments of millimeter scale, requiring a preliminary size reduction step. From its appearance, the residue consisted of hurd (shives), the woody core of the stem, possibly with a small fraction of bast fibers; no treatment other than drying and milling was applied. The skeletal density of the milled hemp, measured by helium pycnometry (AccuPyc II 1340, Micromeritics, Norcross, GA, USA), was 1.444 ± 0.004 g/cm^3^; since it excludes the open lumens of the particles, the volume fractions calculated from it (Section 3.7) are a lower bound. Glacial acetic acid (≥99.7%, Sigma-Aldrich, St. Louis, MO, USA) was used as the casting solvent.

### 2.2. Powder Milling, Film Preparation, and Experimental Design

The hemp was milled to target particle size distributions using a ZM 300 centrifugal mill (Retsch, Haan-Gruiten, Germany) with a 24-tooth rotor, in which size reduction occurs by combined impact, shear, and friction followed by passage through a sieve ring. Sieve sizes of 1, 0.5, 0.25, and 0.08 mm were used to obtain distinct fractions, at a rotor speed of 18,000 rpm. A knife mill (Retsch SM 300), based mainly on cutting, was used for comparison.

**Figure 1:**
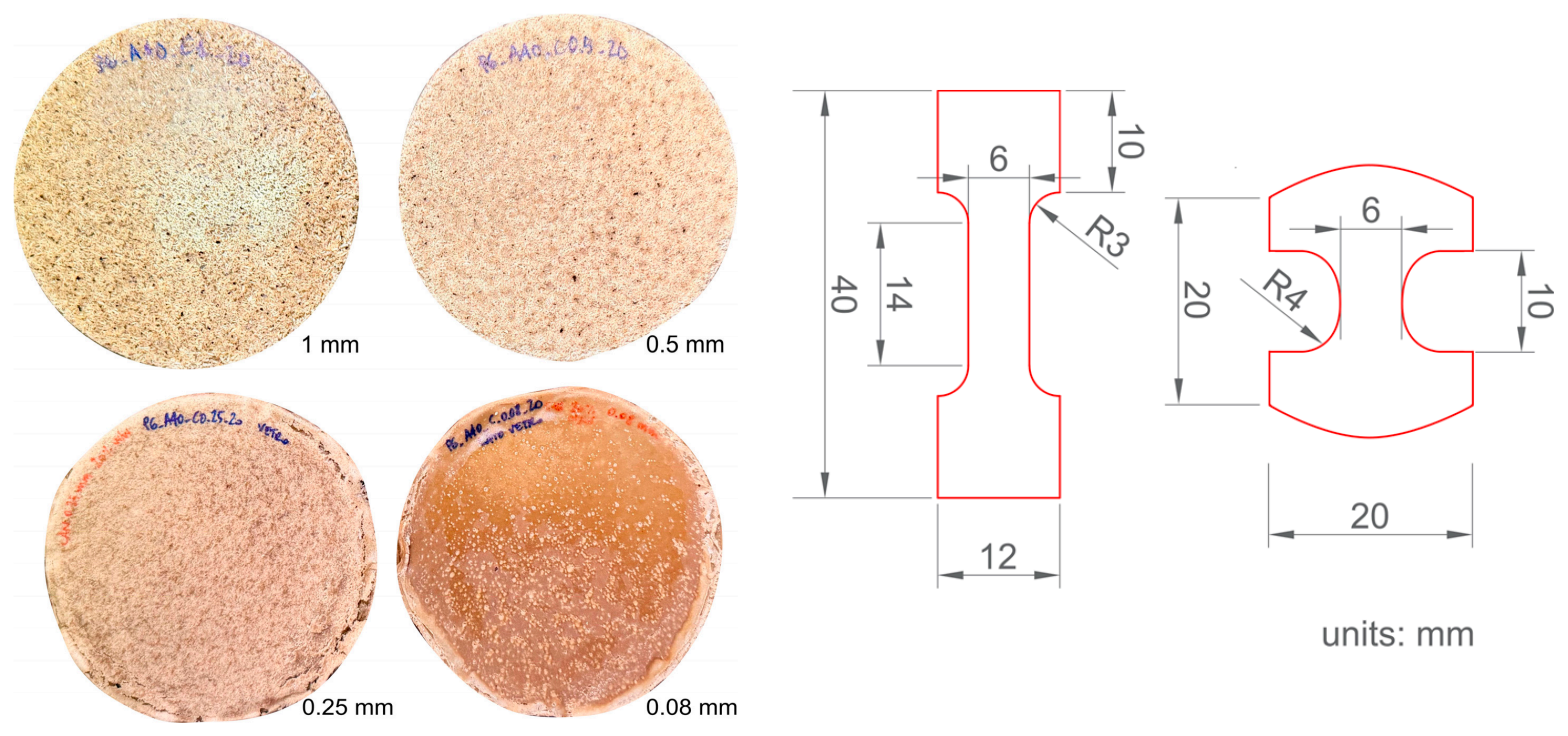
Solvent-cast films and tensile specimens: (**left**) PCL/hemp films with 20 wt% hemp and different particle sizes; (**right**) dimensions (in mm) of the dog bone and butterfly dies (red: die contour).

Films were produced by solvent casting, a preliminary route that yields homogeneous thin films under mild conditions and is well suited to the systematic screening of matrix grade, filler content, and particle size on a lab scale [25,26]. Acetic acid was chosen as a less toxic alternative to chlorinated solvents such as chloroform and dichloromethane and has been shown to dissolve PCL effectively and to be usable in solvent casting and electrospinning [33,34,35,36,37]. Prolonged contact with acidic solvents can promote partial hydrolytic degradation of PCL, to an extent that increases with the contact time [27,28,33,38]. Since the same dissolution conditions were used for all the formulations, a possible degradation is expected to affect mainly the absolute properties rather than the comparison between formulations of the same grade (Section 3.8). For each formulation, 6 g of PCL was dissolved in 40 g of acetic acid (13.0 wt%) at 70 °C under stirring at 300 rpm for 1 h. Films were cast from weighed portions of this solution, 35 g in most cases (23.1 to 45.0 g overall). For composites, hemp powder, previously dried in oven at 80 °C overnight, was added to each portion at the target content, calculated on the mass of the composite (1.50 g and 2.57 g of hemp per 6 g of PCL for 20 and 30 wt%, respectively), and dispersed under manual stirring for 10 min. The PCL concentration in acetic acid was selected empirically to give an initial viscosity high enough to slow down the sedimentation of the particles while the solvent evaporates. The suspensions were poured into Petri dishes with a diameter of 9.6 cm, dried for 24 h on a hot plate at 40 °C inside a fume hood, and then thermally treated at 72 °C for 15 min to improve planarity (Figure 1, left). One film was cast for each formulation, and two for the neat PCL 6800, with different amounts of solution (45 and 35 g) to check the effect of thickness.

The experimental campaign followed a two-stage screening design (Table 1). The filler contents of 20 and 30 wt% were selected on the basis of the intended final application: packaging with a natural appearance that requires a clearly visible filler: lower contents would not provide this appearance, whereas contents above 30 wt% could not be dispersed and cast satisfactorily. Contents in this range are common for lignocellulosic fillers in PCL [29,39], although solution-cast films are usually strongest at lower contents [40], so the selection is dictated by the application rather than by the mechanical optimum. The particle sizes correspond to the sieve rings of the centrifugal mill and bracket the particle-size windows of FDM and injection molding (Section 1). In the first stage, PCL 6500 was used as a broad screening matrix across the four particle sizes at 20 and 30 wt%, plus the neat polymer. In the second stage, PCL 6800 was examined at 20 wt% only, at the three finer sizes (0.08, 0.25, 0.5 mm), plus the neat polymer. PCL 6800 was not tested at 30 wt% because the higher molecular weight produced a more viscous solution that hindered hemp dispersion at the higher loading. The design is therefore intentionally asymmetric, a point taken into account in the grade comparison.

### 2.3. Characterization

Tensile tests were performed on a Z10 universal testing machine (ZwickRoell, Ulm, Germany) equipped with a 1 kN load cell. Specimens were die-cut from the films, mostly in a dog bone geometry (*n* = 72) and, for formulations more sensitive to local defects and breaking upon cutting, in a butterfly geometry (*n* = 14) (Figure 1, right). The gauge length was 20 mm for dog bone and 10 mm for butterfly specimens. Tests were performed at 1 and 0.5 mm/min, respectively, giving the same nominal strain rate of 0.05 min^−1^ in the two geometries. The dies were not manufactured according to a specific standard: non-standard geometries were adopted because of the limited size of the solvent-cast films and because some formulations tended to break during cutting [41]. The average thickness was approximately 1 mm and the width approximately 6 mm. Elastic modulus, tensile strength, and strain at strength were extracted from the stress–strain curves. Between 4 and 12 specimens were tested per formulation, all cut from the same film, so the replicates describe the variability within a film rather than between casts; values are reported as mean ± 1 standard deviation (SD). The thickness of each specimen was measured with a micrometer. The two casts of neat PCL 6800, prepared with different amounts of solution to check the effect of thickness, differed by about 14% in mean strength and modulus, but only by 3% and 5% after thickness correction with Equation (1) (Welch t-test, *p* = 0.56 and 0.38), indicating that thickness is the main source of variation between casts. The films were not perfectly uniform, due to small irregularities of the Petri dishes and an imperfect leveling of the hot plate, and the measured thickness exceeded that of a dense, uniform film calculated from the poured masses by 1.2 to 1.6 times for the neat films and the 0.08 and 0.25 mm fractions at 20 wt%, and by 2.0 to 3.0 times for the 0.5 and 1.0 mm fractions and at 30 wt% (Figure A4).

**Figure A1:**
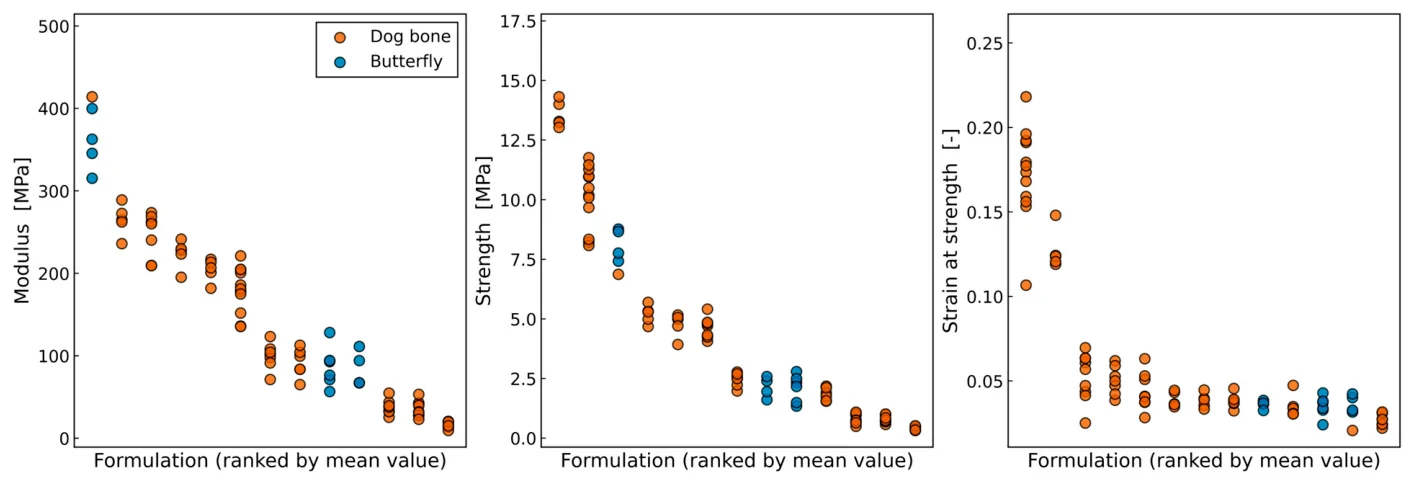
Effect of tensile specimen geometry on modulus (**left**), strength (**center**) and strain at strength (**right**). Each point is one specimen, grouped by formulation; clusters are ordered by the mean of the property shown in each panel. Dog bone and butterfly distributions overlap for modulus and strength but are offset for strain, consistent with the ANCOVA of Section 2.3.

Because two geometries were used, a multi-factor analysis of covariance (ANCOVA) on log-transformed properties (adopting formulation and geometry as factors) was used to check for a systematic geometry effect. No significant effect was found for modulus (*F* = 0.48, *p* = 0.49) or strength (*F* = 0.75, *p* = 0.39), supporting the pooling of the two geometries for these properties, whereas a significant effect was found for strain at strength (*F* = 7.38, *p* = 0.008), which is therefore discussed only qualitatively. Group comparisons used one-way ANOVA followed by Bonferroni-corrected Welch t-tests; two-group comparisons used Welch t-tests. The distribution of the individual specimens by geometry is shown in Figure A1, where specimens are grouped by formulation and the formulation clusters are ordered by the mean value of the property displayed in each panel. Statistical analyses and the model fitting were carried out in Python (v3.12) with the SciPy (v1.11) and statsmodels (v0.15) libraries.

The particle size distribution of the milled hemp was measured by laser diffraction (Microtrac Sync, Microtrac MRB, Haan, Germany) [42], used as an indicative check of the size reduction. Distributions were obtained for the four centrifugal mill fractions used in the composites and, for comparison, for the two coarse knife mill fractions.

Polished cross-sections of five representative formulations were examined by scanning electron microscopy. Specimens were embedded in EpoThin 2 epoxy resin (Buehler, Lake Bluff, IL, USA), then ground and polished on a Mecatech 334 machine (Presi, Eybens, France) to a 1 μm finish under gentle conditions (plate and specimen holder co-rotating at 80 and 60 rpm, respectively, 1 N acting on each specimen, water as lubricant and coolant) to limit the deformation of the ductile matrix as much as possible, and gold sputter-coated (Edwards Sputter Coater S150B, Burgess Hill, UK) to limit charging. Images were taken with a MIRA 3 scanning electron microscope (TESCAN, Brno, Czech Republic) at an accelerating voltage of 10 kV and a working distance of 15 mm. The formulations imaged were PCL 6500 and PCL 6800 with 20 wt% hemp at 0.08 and 0.25 mm, which compare the two grades at the two finest sizes, together with PCL 6500 with 30 wt% hemp at 0.25 mm.

The thermal behavior of the matrices was analyzed on the as-received pellets and on the neat solvent-cast films of both grades (for PCL 6800, the film cast from 45 g of solution), one sample per material. Differential scanning calorimetry (DSC) was carried out with a DSC 250 calorimeter (TA Instruments, New Castle, DE, USA) under nitrogen (50 mL/min), on samples of about 15 to 20 mg in Tzero aluminum pans. The thermal program consisted of a heating from −90 to 110 °C, a cooling to −90 °C, and a second heating to 110 °C, followed by a second cooling, all at 10 °C/min, with 5 min isotherms at 110 and −90 °C. The first heating describes the films after storage at room temperature, whereas the cooling and the second heating, recorded after the thermal history had been erased, compare the materials themselves. The melting and crystallization temperatures (*T_m_* and *T_c_*) were taken at the peaks, and the corresponding enthalpies (Δ*H_m_* and Δ*H_c_*) were obtained by integration over a sigmoidal baseline drawn between the tangents to the solid (−40 to −25 °C) and melt regions (92 to 102 °C on heating, 50 to 85 °C on cooling), with the same limits for all the samples. The degree of crystallinity was calculated as *X_c_* = Δ*H_m_*/Δ*H_m_*^0^, with Δ*H_m_*^0^ = 148 J/g for 100% crystalline PCL [27]. The glass transition temperature (*T_g_*) was taken at the midpoint of the heat capacity step on heating, and the heat capacity change (Δ*c_p_*) as the step height divided by the heating rate.

Thermogravimetric analysis (TGA) was carried out on the same materials with an SDT Q600 analyzer (TA Instruments, New Castle, DE, USA). Samples of about 15 to 35 mg were placed in open alumina crucibles and heated from 30 to 600 °C at 10 °C/min under nitrogen (100 mL/min). The temperature at 5% mass loss (*T*_5%_) and the temperature of the maximum mass loss rate (*T_max_*, peak of the derivative curve) were determined, and the mass loss up to 150 °C was taken as a measure of the volatile content (moisture and residual solvent).

## 3. Results and Discussion

### 3.1. Particle Size Distribution and Selection of the Milling Method

**Figure 2:**
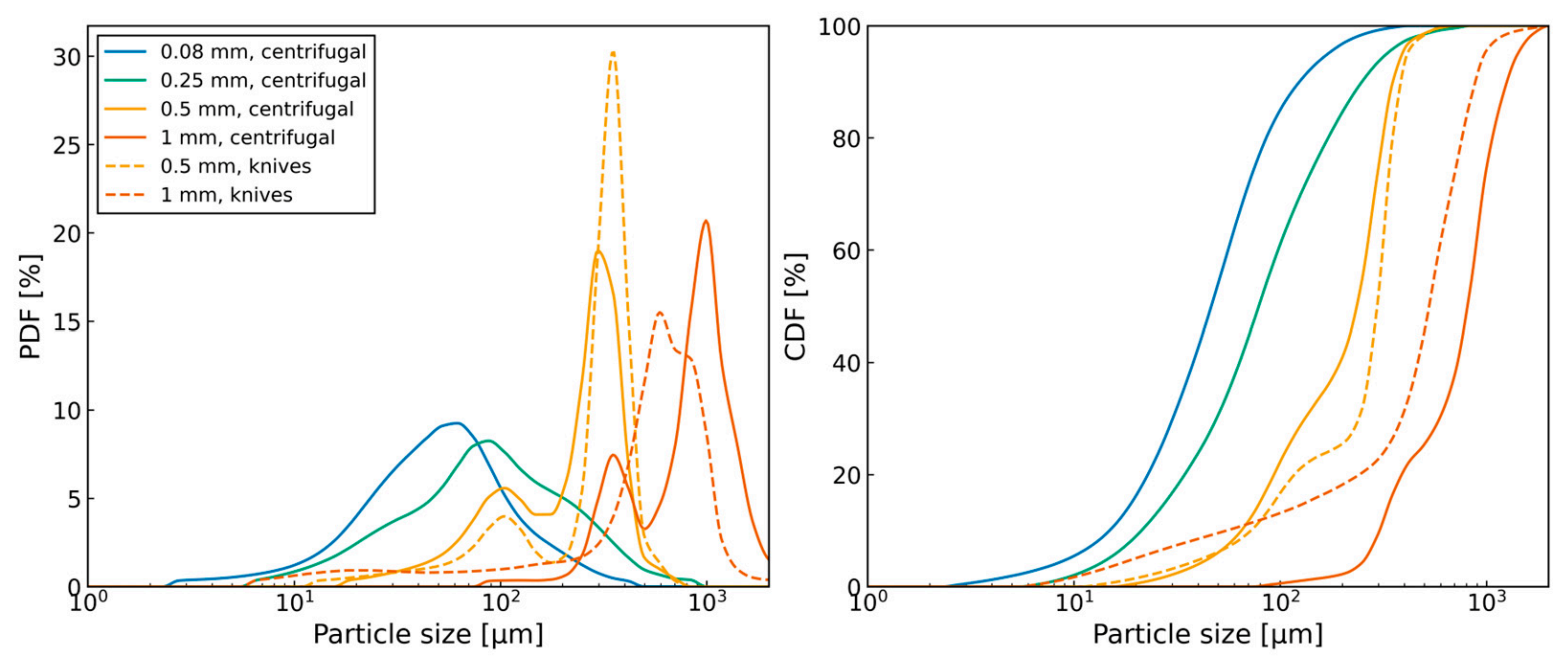
Particle size distribution of hemp powders obtained using different milling techniques. Probability (**left**) and cumulative (**right**) density functions.

The particle size distributions of the milled hemp are shown in Figure 2, and the corresponding characteristic diameters are listed in Table 2. For the centrifugal mill, the distributions shifted regularly toward larger sizes as the sieve opening increased, with a median diameter D50 rising from about 46 micrometers at the 0.08 mm sieve to about 809 micrometers at the 1.0 mm sieve. The knife mill, available only at the 0.5 and 1.0 mm sieves, gave less regular distributions and was clearly bimodal at 1.0 mm (Figure 2). Since laser diffraction reports an equivalent spherical diameter and the hemp particles are fibrous, the values in Table 2 are indicative rather than exact.

The centrifugal mill was selected for composite preparation for two reasons. At the sieve sizes common to both mills, it produced more regular, single-peaked distributions, whereas the knife mill did not. More decisively, it was the only mill able to reach the fine 0.08 and 0.25 mm fractions required for the composites and compatible with one of the intended final routes (FDM). The centrifugal mill was therefore used for all composite formulations.

### 3.2. Microstructure of the Cast Films

**Figure 3:**
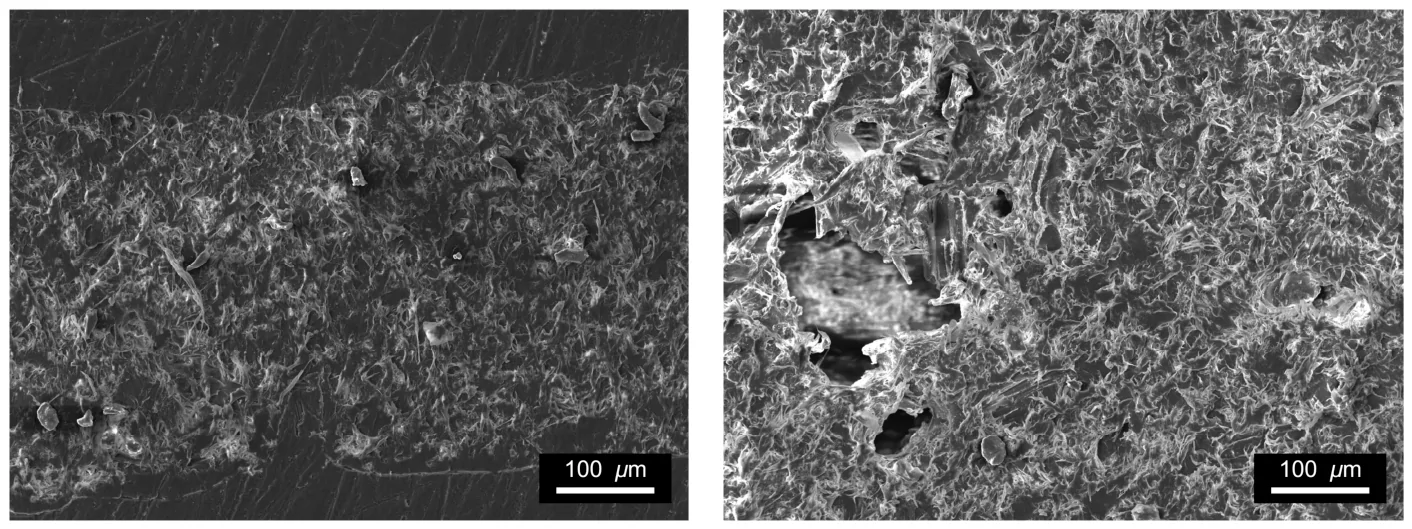

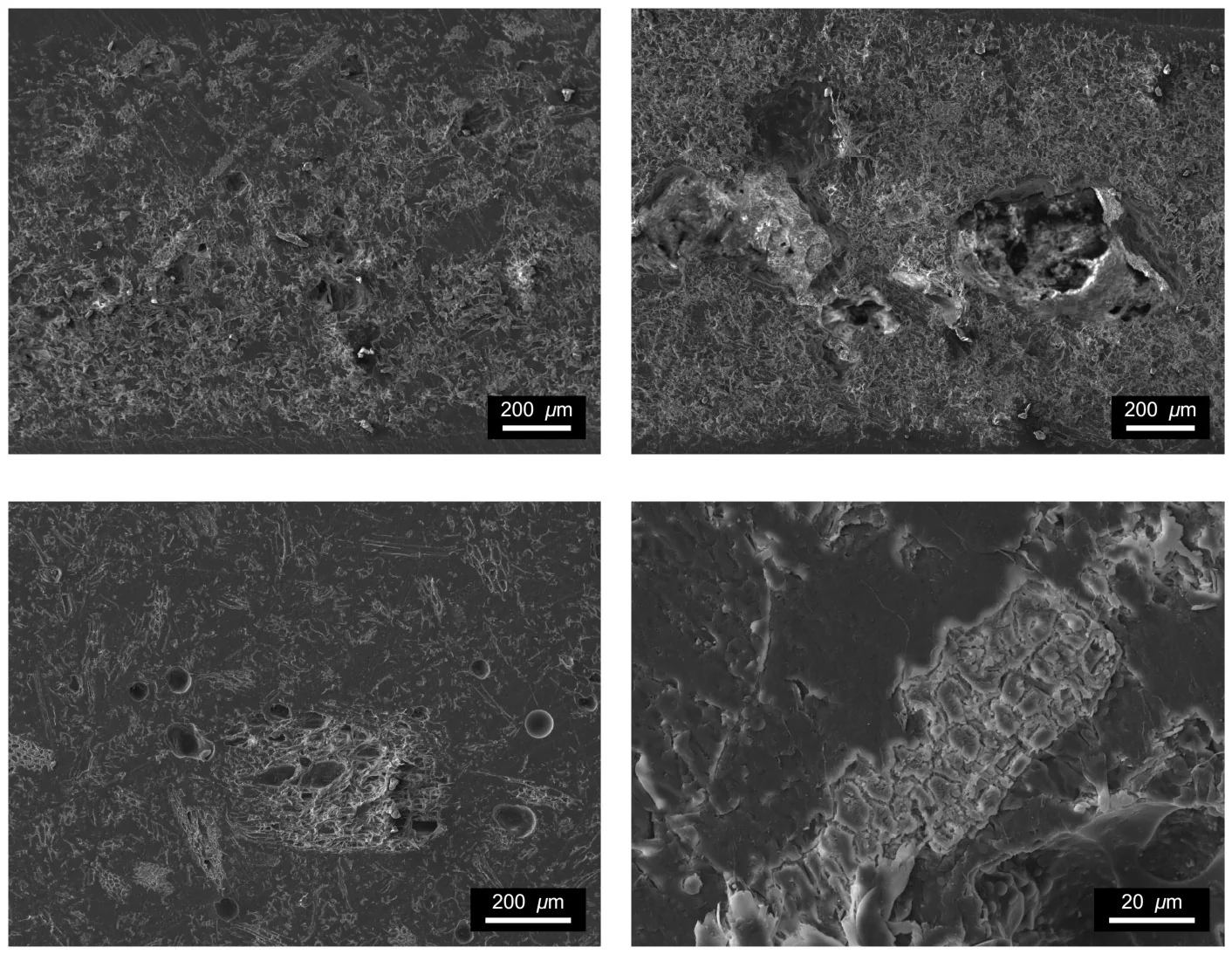
SEM of polished cross-sections of solvent-cast PCL/hemp composites. (**top**, **left**) PCL6500_20_0.08, (**top**, **right**) PCL6800_20_0.08, (**mid**, **left**) PCL6500_20_0.25, (**mid**, **right**) PCL6800_20_0.25, (**bottom**, **left**) PCL6500_30_0.25, (**bottom**, **right**) PCL6500_20_0.25. Magnification: 1000× (**top row**), 400× (**mid row**), 500× (**bottom**, **left**), and 5000× (**bottom**, **right**).

Cross-sectional scanning electron microscopy showed the microstructure resulting from the solvent casting process. Local deformation features of the matrix introduced by polishing are visible in some micrographs. Grinding and polishing a ductile matrix can hide defects, when the matrix flows over voids and interfacial gaps, but can also introduce them, when particles are pulled out or the matrix is torn. The micrographs were therefore interpreted only qualitatively, and the features that could also result from the preparation are indicated as such. Porosity was present in almost all formulations, on more than one length scale and had more than one origin. The matrix contained roughly spherical bubbles and irregular voids (Figure 3, top right and mid right). The bubbles, whose rounded shape differs from that of the cavities left by pulled-out particles, are attributable to air entrapped during mechanical stirring and to vapor that nucleates and grows beneath the drying skin as the solvent leaves [43]; the irregular voids can have the same origin, but some of them may also be cavities left by particles pulled out during polishing. Separately, the hemp particles carry their own cellular, lumen-based porosity (Figure 3, bottom left), which is intrinsic to the filler and represents a larger share of the section for the coarser fractions. The finest, lowest-content film (PCL 6500, 20 wt%, 0.08 mm) was essentially free of matrix voids (Figure 3, top left). Voids of both kinds act as stress concentrators and crack initiation sites, consistent with the low Weibull moduli reported in Section 3.4 and with the defect-controlled reading of the mechanical data. Dispersion quality was found to be dependent on particle size. At 0.08 mm the fine hemp was uniformly distributed in both grades (Figure 3, top row), whereas at 0.25 mm the distribution was less uniform and left pockets of neat matrix between hemp-rich regions (Figure 3, mid row). This is consistent with the drying kinetics of particle-filled films, in which the tendency of particles to remain uniformly distributed during solvent evaporation decreases as particle size increases [44,45,46], and it provides a microstructural basis for the mechanical size effect of Section 3.4: fine particles are well distributed and can redistribute load, whereas coarser particles leave unreinforced matrix pockets and concentrate stresses.

The two PCL grades differed in their void content. At matched size and loading, PCL 6800 showed more and larger bubbles and voids than PCL 6500. A plausible explanation links this to the matrix molecular weight: the higher molecular weight PCL 6800 forms a more viscous casting solution, which slows both the escape of entrapped air and the diffusion of solvent to the surface, favoring early skin formation and the growth of trapped vapor bubbles [43]. Casting solution viscosity was not measured, so this remains a qualitative observation, but it is a processing–structure–property link tied to a polymer variable and it helps explain why the higher grade does not translate its potential into a proportional mechanical benefit. Finally, micrographs suggested the presence of a weak filler/matrix interface. At high magnification the hemp particles were separated from the matrix by a continuous gap (Figure 3, bottom right). Since a gap at the interface can also open during polishing, this observation does not prove debonding on its own, but it is consistent with the poor adhesion indicated by the tensile results, in which hemp acted mainly as a filler and reduced the strain at strength (Section 3.5).

### 3.3. Thermal Properties

**Figure 4:**
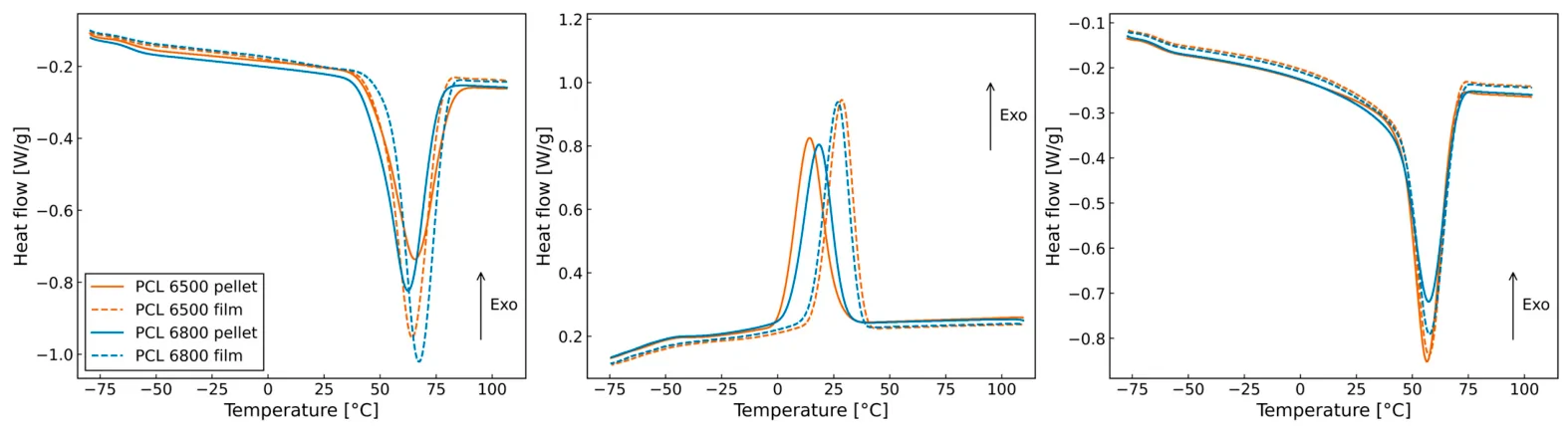
DSC curves of the as-received pellets (solid lines) and of the neat solvent-cast films (dashed lines) of PCL 6500 and PCL 6800 at 10 °C/min: (**left**) first heating, (**center**) cooling, (**right**) second heating. Exothermic up.

The DSC curves of the as-received pellets and of the neat cast films are shown in Figure 4, and the main parameters are listed in Table 3. In the first heating, the films were more crystalline than the pellets (*X_c_* = 52.2 versus 44.4% for PCL 6500 and 52.7 versus 45.5% for PCL 6800). Much of this difference reflects the different thermal history of the two forms: the films were briefly heated above their melting range during the planarity treatment at 72 °C (Section 2.2), then solidified and stored at room temperature, whereas the pellets retain the history of their industrial production. Since all the films received the same treatment, this history does not affect the comparison between formulations. Once the thermal history was erased, the differences were smaller (*X_c_* = 44.0 versus 41.2% for PCL 6500 and 42.5 versus 38.8% for PCL 6800 in the second heating), and the melting peaks coincided (56.7 to 57.9 °C). Casting had a larger effect on crystallization from the melt: on cooling, the crystallization peak of the films was 14.9 °C (PCL 6500) and 8.9 °C (PCL 6800) higher than that of the pellets, and the second cooling reproduced these values within about 1 °C. The glass transition was not affected (*T_g_* = −63.8 to −63.0 °C in the second heating for all the samples), while the heat capacity step at *T_g_* was smaller for the films, consistent with their higher crystallinity.

These values are in line with those reported for PCL. The glass transition temperature agrees with the −65 °C measured on Capa 6500 [31]. Skoglund and Fransson [47] obtained the same *T_g_* for PCL with number-average molecular weights between 22 and 116 kg/mol, with a heat capacity step of about 0.1 J/(g·K) and melting enthalpies of 57 to 70 J/g, comparable with those measured here in the second heating (57.4 to 65.0 J/g); they also found that the crystallization temperature decreases with increasing molecular weight. An increase in crystallization temperature similar to that observed here was reported by Gil-Castell et al. for Capa 6800 dissolved in formic/acetic acid, from 21.3 °C for the pellets to 27.4 °C after 24 h, together with an increase in crystallinity and a marked reduction of molar mass [27]; a loss of molar mass during dissolution was also found in acetic/formic acid 9:1 [28]. The higher crystallization temperature and crystallinity of the cast films are therefore consistent with the partial hydrolytic degradation during dissolution anticipated in Section 2.2. Its extent cannot be quantified by DSC, since the crystallization temperature of commercial PCL also depends on heterogeneities such as catalyst residues [48]. In any case, all the formulations of a grade were prepared under the same conditions, so any possible degradation affects their absolute properties rather than the comparison between them.

**Figure 5:**
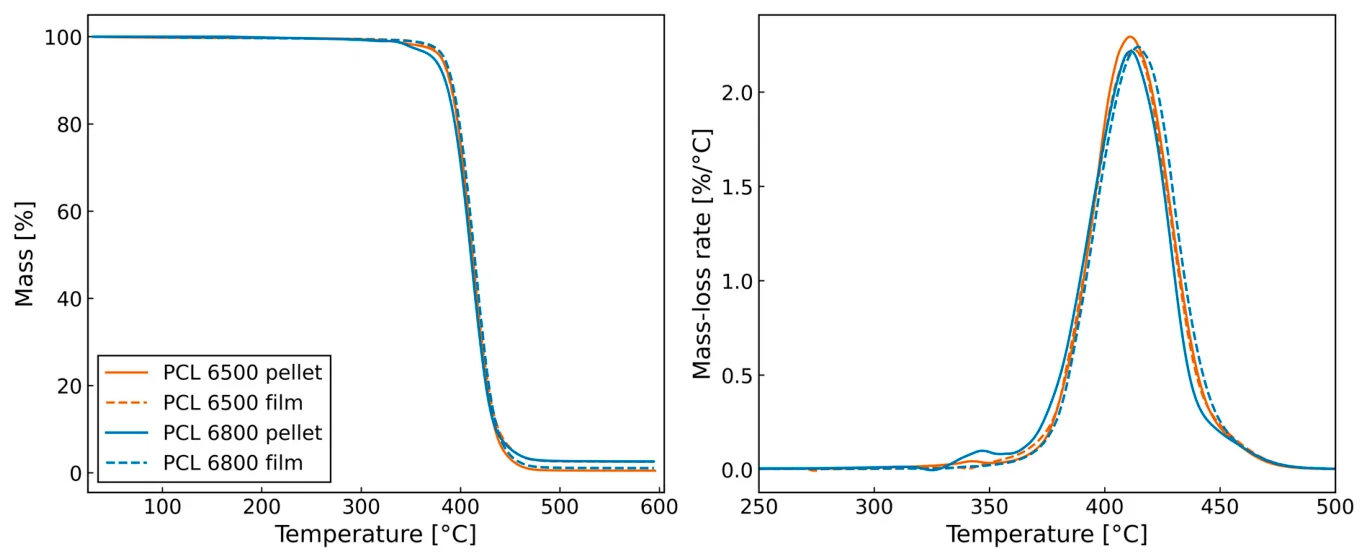
TGA curves of the as-received pellets (solid lines) and of the neat solvent-cast films (dashed lines) of PCL 6500 and PCL 6800 under nitrogen at 10 °C/min: (**left**) mass, (**right**) mass loss rate.

The TGA curves (Figure 5) showed a single main decomposition step for all the samples, with the maximum mass loss rate at 411.0 to 414.6 °C, in agreement with the values reported for high molecular weight PCL in nitrogen at the same heating rate [39,49]. Casting did not reduce the thermal stability: the temperatures at 5% mass loss and at the maximum rate of the films were equal to or slightly higher than those of the pellets (*T*_5%_ = 381.0 °C for both PCL 6500 samples, 383.9 versus 373.1 °C for PCL 6800). The pellets also showed a small additional mass loss at about 345 °C (less than 2% of the mass), which lowered the *T*_5%_ of the PCL 6800 pellets and was absent in the films. Since a lower molecular weight lowers the decomposition temperature of PCL [50], and low molecular weight PCL decomposes in more steps starting from about 280 °C [49], the TGA shows no sign of an extensive degradation, and the degradation suggested by the DSC was therefore limited. The mass loss up to 150 °C was below 0.3% for all the samples, films and pellets alike, so no significant residual solvent remained in the films after storage at room temperature. Acetic acid can continue to diffuse out of the films during storage, so this value, like the first DSC heating, describes their long-term state and does not exclude a higher solvent content in the freshly cast films.

### 3.4. Tensile Behavior of the Neat Matrices and Defect-Controlled Response

**Figure 6:**
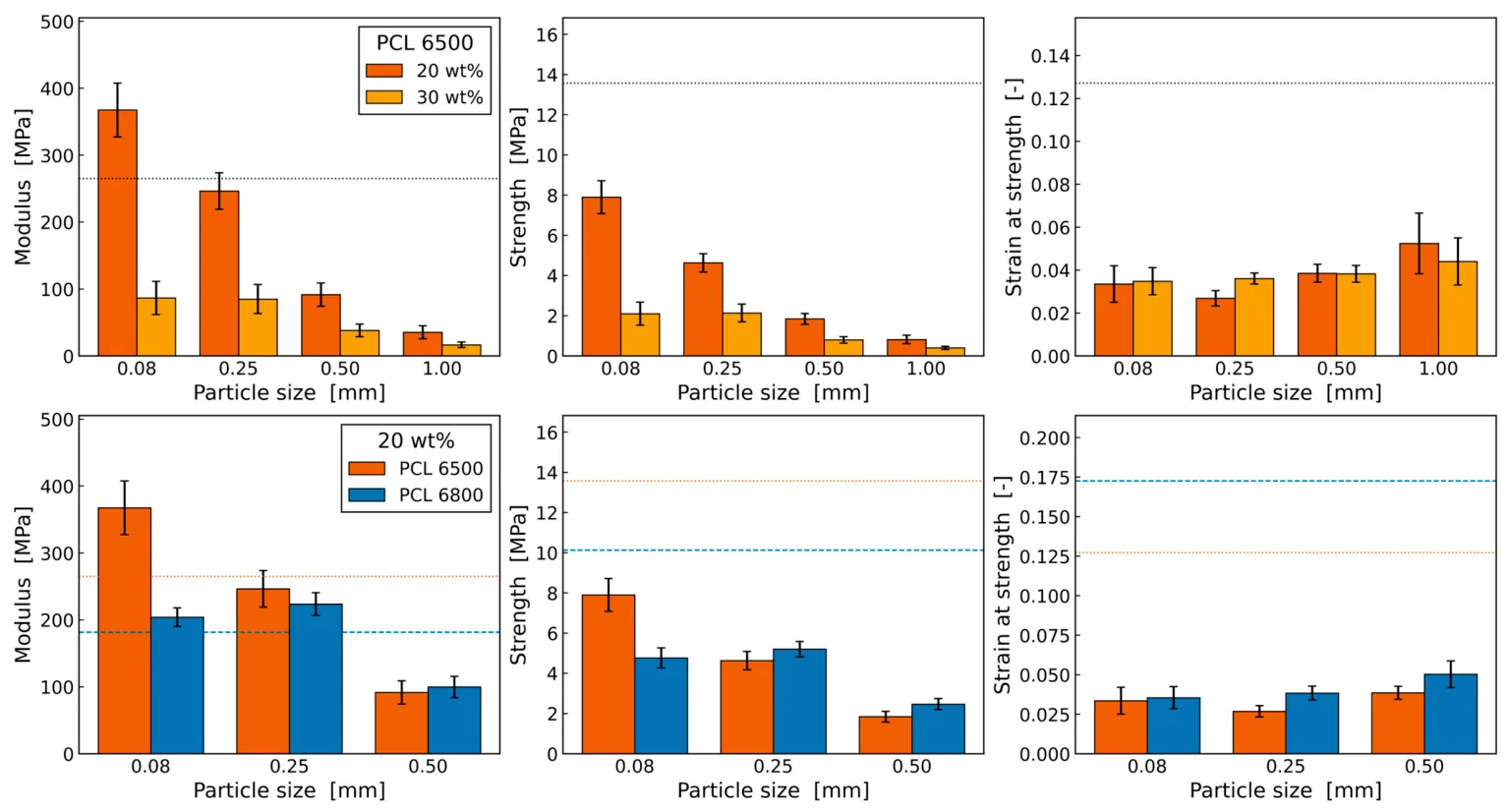
Tensile properties (mean ± SD). (**top**) PCL 6500 at 20 and 30 wt% across particle sizes; (**bottom**) PCL 6500 versus PCL 6800 at 20 wt%. Dotted lines indicate neat polymer values.

The two neat matrices differed significantly in all properties (Figure 6). PCL 6500 was stiffer and stronger than PCL 6800 (*E* = 265 ± 19 versus 182 ± 28 MPa, *p* < 0.001; *σ* = 13.6 ± 0.6 versus 10.1 ± 1.3 MPa, *p* < 0.001) and less ductile (strain at strength 0.127 ± 0.012 versus 0.173 ± 0.028, *p* < 0.001). This can be attributed to the lower molecular weight of PCL 6500, which favors higher chain mobility and crystallization, therefore producing a stiffer, stronger, and less ductile film, whereas the higher entanglement density of PCL 6800 favors deformability [48,51,52,53]. After storage at room temperature, the two neat films showed the same crystallinity (Section 3.3). This is not in contrast with the explanation above: PCL continues to crystallize at room temperature for weeks after solidification, with a corresponding increase in modulus and yield stress [54], and the higher molecular weight grade crystallizes more slowly and to a lower extent [47,52]. The two grades can therefore approach similar crystallinity in the long term while differing at earlier stages, so the values measured after storage represent the long-term state of the neat films rather than a direct measure of the difference between them at the time of the tensile tests.

**Figure 7:**
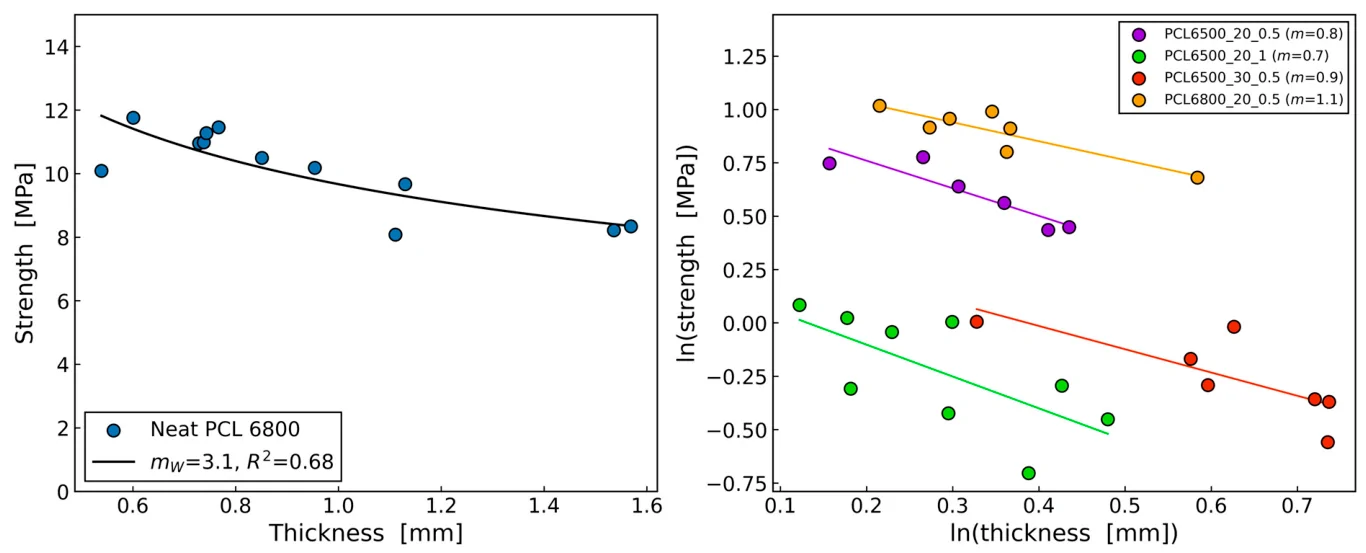
Weibull thickness scaling of strength: (**left**) neat PCL 6800 (*m_W_* = 3.1); (**right**) log-log weakest link fits within composite formulations.

The absolute values are lower than those usually reported for bulk PCL, particularly the strain at strength. This is most likely associated with the solvent-cast route and the die-cutting of specimens, which introduce residual solvent, thickness heterogeneity and local stress concentrations as possible detrimental factors [27,28]. The sensitivity of thin-film tensile measurements to specimen geometry is a further factor [41]. In line with defect-controlled behavior, the strength of the neat PCL 6800 films decreased with increasing thickness following a Weibull weakest link power law, $\sigma \propto t^{-1/m}$, with a low Weibull modulus *m_W_* ≈ 3.1 (*R*^2^ = 0.68, *p* = 0.001) (Figure 7, left) [55]. This relation was obtained by plotting the natural logarithm of strength against the natural logarithm of thickness: the slope of the linear regression equals −1/*m*, so the Weibull modulus *m* is the negative reciprocal of the slope, and a low value denotes a broad, defect-sensitive strength distribution. The same construction was applied within individual composite formulations, each fitted separately (Figure 7, right), where the slopes (*m* ≈ 1) indicate an even higher defect sensitivity than the neat polymer. The mechanical characterization is therefore best read as a comparative screening of relative differences between formulations; the absolute values should not be taken as intrinsic properties of the matrices.

### 3.5. Effect of Filler Content and Particle Size

In the case of PCL 6500 composites, increasing the filler content from 20 to 30 wt% reduced both strength and modulus at every particle size (Figure 6, top). The strength dropped by a factor of about 2.5 to 4 depending on size (from 7.9 ± 0.8 to 2.1 ± 0.6 MPa at 0.08 mm and 1.8 ± 0.3 to 0.8 ± 0.2 MPa at 0.5 mm; all comparisons *p* < 0.001). An ANCOVA including log(thickness) as a covariate confirmed that this reduction is largely independent of the thickness increase associated with the more viscous 30 wt% solutions, the filler-content effect remaining significant at 0.25, 0.5, and 1.0 mm (*p_adj_* < 0.05) and becoming marginal only at 0.08 mm (*p_adj_* = 0.076). Under these conditions, hemp therefore behaves as a filler, with little reinforcing effect. The additional filler reduces the continuity of the load-bearing matrix and limits stress transfer, in agreement with reports on particulate lignocellulosic/PCL systems [18,19]. The strain at strength was also reduced relative to the neat polymer. Together with the decrease in strength, this indicates poor interfacial adhesion between hemp and PCL, which prevents an effective stress transfer to the particles at large strains, consistent with the interfacial gaps observed by SEM (Section 3.2).

Particle size had a strong effect on both strength and modulus (one-way ANOVA *p* < 0.0001 for all grade and content combinations). Instead of a single optimum, the properties showed a plateau at the fine end followed by a step decrease. For PCL 6500 at 30 wt% and for PCL 6800 at 20 wt%, the 0.08 and 0.25 mm fractions were statistically indistinguishable in both strength and modulus (*p_adj_* > 0.25), with a marked drop at 0.5 mm and a further drop at 1.0 mm (*p_adj_* < 0.01). Finer particles likely promote better dispersion and reduce the size of the strength-controlling defects, whereas coarse particles act as stress concentrators and crack initiation sites [23]. In the solvent-cast films, the particles of the 1.0 mm fraction are as large as the film (D90 of 1.25 mm, Table 2, against a dense-film thickness of 0.45 to 0.76 mm), so they protrude from the surface and set the film thickness (Figure A4). Since thin films are preferable in solvent casting to let the solvent escape, this limitation is intrinsic to coarse particles in this route. The films with the 0.5 mm fraction and those at 30 wt% were also thicker than a dense film, although their particles are smaller than the film; here the excess thickness reflects the porosity introduced by the filler and the incomplete filling of the particle packing by the drying solution. The data therefore indicate that reducing the size from 0.5 to 0.25 mm markedly improves the properties, while a further reduction to 0.08 mm gives no additional gain. From the perspective of the intended processing routes, the plateau at the fine end is convenient, since the finest fractions are also those compatible with FDM, where the nozzle diameter would in any case require a small particle size. For injection molding, which tolerates coarser powder, the same relationship quantifies the mechanical penalty associated with moving toward larger particles.

### 3.6. Effect of Matrix Grade and Modulus Reinforcement at Fine Sizes

At 20 wt%, the grade ranking was particle size-dependent (Figure 6, bottom). At 0.08 mm, PCL 6500 was clearly superior in both strength and modulus (*p* < 0.001). At 0.25 and 0.5 mm, the modulus of the two grades was statistically equivalent, while the strength was higher for PCL 6800 (*p* = 0.04 at 0.25 mm; *p* = 0.002 at 0.5 mm) and the strain at strength was consistently higher for PCL 6800. A plausible interpretation is that the higher entanglement density of PCL 6800 helps to bridge the larger defects associated with coarser particles, whereas at the finest size the higher intrinsic stiffness of PCL 6500 dominates. This interpretation is consistent with the molecular weight behavior of the neat matrices but remains tentative, since the molecular weight after casting was not measured and a different extent of degradation of the two grades in acetic acid cannot be excluded (Section 3.8).

For PCL 6800 at 20 wt%, the modulus at the two finest sizes was significantly higher than that of the neat polymer (204 ± 14 MPa at 0.08 mm, *p* = 0.045; 224 ± 17 MPa at 0.25 mm, *p* = 0.003; versus 182 ± 28 MPa for the neat polymer). Under these conditions hemp behaves as a mild reinforcing phase for the modulus, an exception to the general filler behavior that is not observed for the strength, which always decreases with filler addition. A comparable distinction between a modulus increase and a strength decrease on adding a rigid lignocellulosic filler has been reported for bamboo powder/PCL [19] and, for hemp fibers in the same matrix, as a modulus gain without a strength gain [18]. The opposite responses of modulus and strength reflect the different quantities they probe. The strength is failure-controlled and set by the worst defect or stress concentrator, so a rigid, poorly bonded particle almost always lowers it. The modulus is a small-strain average over the whole specimen and depends on the volume-weighted stiffness of the phases and on matrix continuity, so a stiff, well-dispersed fine particle can raise it before the loss of matrix continuity at higher loading or coarser sizes prevails. In the present system, this balance tips toward reinforcement only for PCL 6800 at the two finest sizes, where the more entangled matrix sustains load transfer to well-dispersed particles, whereas for PCL 6500 the thickness-corrected data shows no net modulus gain. The same asymmetry is reflected in the model of Section 3.7, where the strength is described by a decreasing filler term alone while the PCL 6800 modulus requires an additional reinforcement term.

### 3.7. Quantitative Model of the Filler and Defect Contributions

To put the trends on a quantitative basis, a combined model was fitted to the thickness-corrected data. The model is not intended as a predictive law. Its purpose is to separate two effects that are confounded in the raw data: the filler effect (particle size and content) and the thickness/defect scaling introduced by the solvent-cast route. The analysis of variance establishes that these effects are significant; the model quantifies them separately and makes the filler coefficient comparable across particle sizes, which the analysis of variance alone cannot do. In details, the strength and modulus were described by a Pukanszky-type exponential filler term combined with a Weibull weakest link thickness factor:

(1) $$P=\;P_{m}exp\left(-a\varphi^{q}\right){\left(t/t_{ref}\right)}^{-1/m}$$

where *P* is the property (strength or modulus), *φ* the hemp volume fraction (0.160 and 0.246 at 20 and 30 wt%, Section 2.1), *t* the specimen thickness, *t_ref_* = 1 mm a reference value, *P_m_* the relevant matrix property, *a* a size-dependent filler coefficient, *q* the filler content exponent and *m* the thickness-scaling exponent [55,56,57,58]. The exponent *m* was fitted independently for each property and grade and is not transferred between them: for strength it coincides with the Weibull modulus of the weakest link analysis of Section 3.4 (*m* ≈ 3.0 for PCL 6500), whereas for the modulus, which is not failure-controlled, the same thickness factor is retained on a purely empirical basis and takes a different value (*m* ≈ 1.4 for PCL 6500). The exponential form is the no-adhesion limit of the Pukanszky model and remains non-negative over the whole composition range. The content exponent *q* is only weakly identified by the present data, because *a* and *q* are strongly correlated and the objective function is shallow along the *q* direction, so a wide range of *q* gives almost equivalent fits. To keep *a* interpretable, *q* was fixed at values consistent with the non-adhesion particulate composite literature (1.31 for strength, 2.16 for modulus) [56,58], and only the size-dependent coefficient *a* was fitted; using a freely fitted *q* instead changes the absolute *a* values but not the trends or the quality of fit.

**Figure 8:**
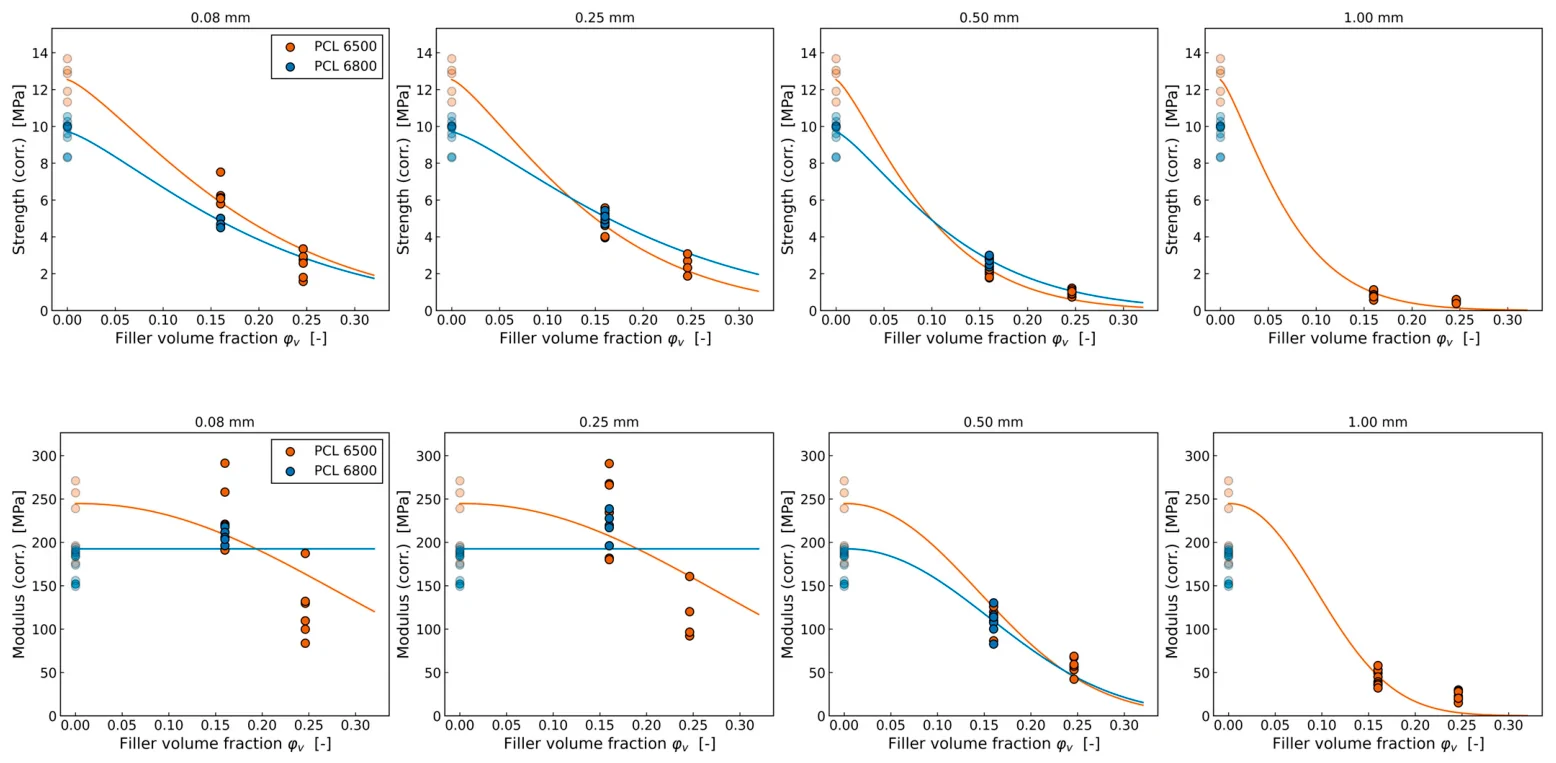
Combined-model fits, one panel per particle size, thickness-corrected to 1 mm: (**top**) strength; (**bottom**) modulus. Solid curves are the size-dependent exponential model; PCL 6800 is omitted at 1.0 mm (not tested).

**Figure A2:**
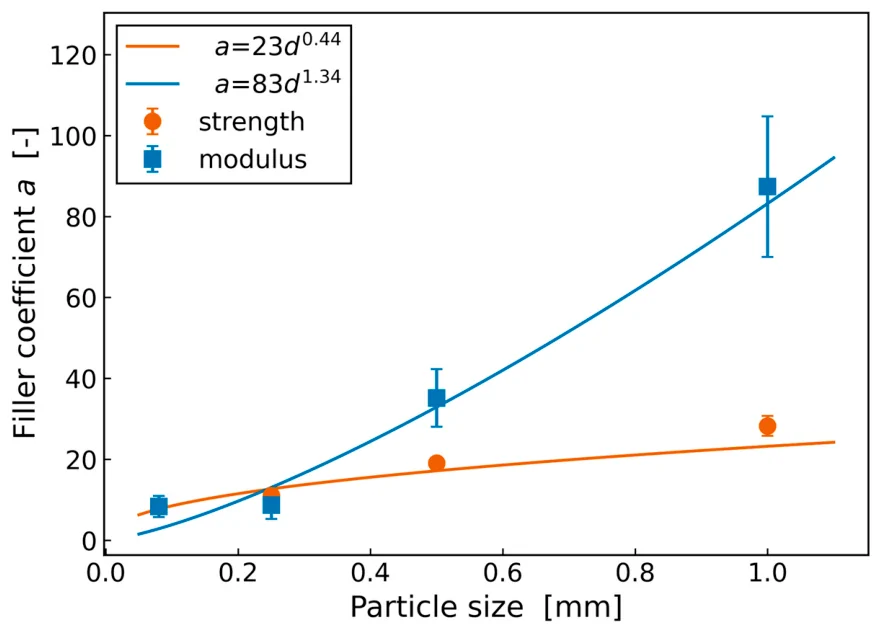
Dependence of coefficient a from Equation (1) on particle size.

**Figure A3:**
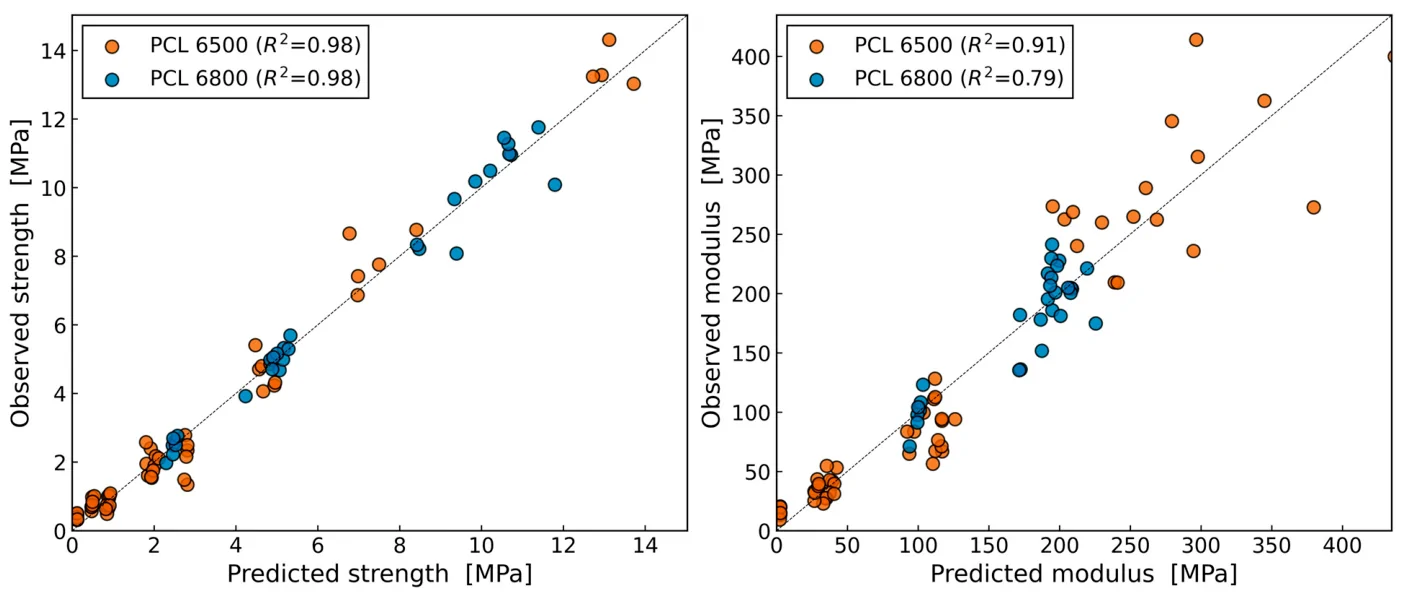
Comparison of observed and predicted values of strength (**left**) and modulus (**right**). Predictions were obtained using Equation (1). The dashed line is the 1:1 reference.

For PCL 6500, the model described the strength very well (*P_m_* = 12.5 MPa, *m* = 3.0, *R*^2^ = 0.979, *RMSE* = 0.56 MPa) and the modulus reasonably well (*P_m_* = 245 MPa, *R*^2^ = 0.907) (Figure 8). The coefficient *a* increased monotonically with particle size, from 8.4 at 0.08 mm to 28.3 at 1.0 mm for the strength, following an approximate power law $a\left(d\right)\propto d^{0.44}$ (Figure A2). A larger coefficient corresponds to a steeper property loss per unit filler, in line with the more severe stress concentration introduced by coarser particles [23]. The low fitted Weibull modulus (*m* ≈ 3 for strength) is consistent with the independent within-formulation Weibull fits of Section 3.4 and supports the residual solvent and defect population interpretation of the reduced absolute properties. Observed and predicted values are compared in Figure A3 and confirm the quality of the fit; the values and predictions reported in the plot, including those for the modulus, were obtained with Equation (1).

**Figure 9:**
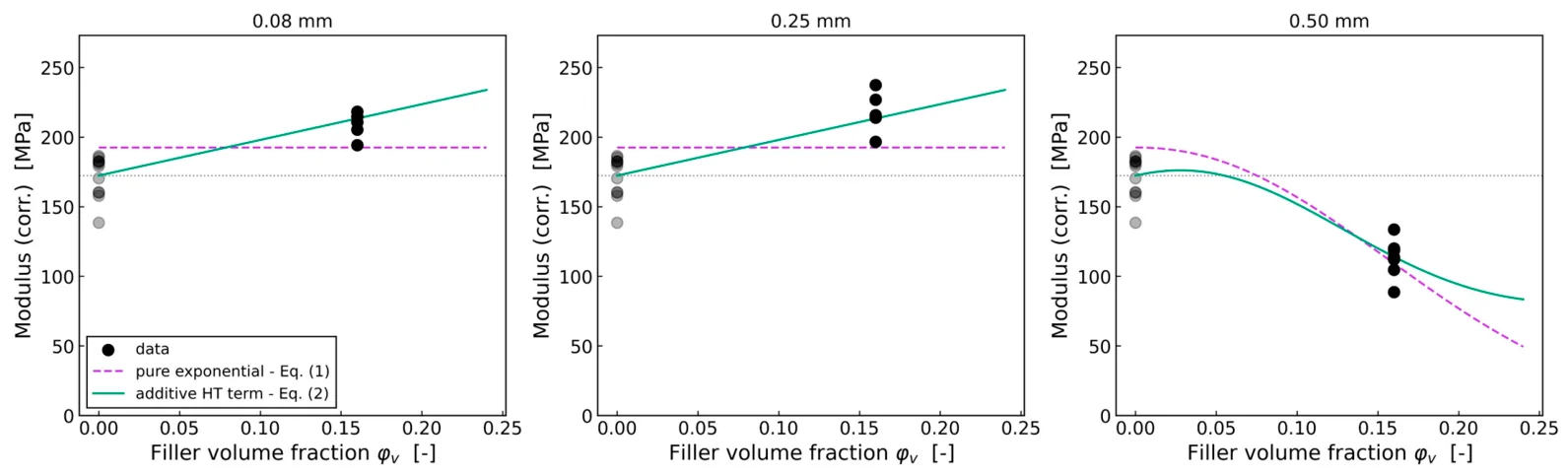
PCL 6800 modulus: the additive Halpin–Tsai term (solid) captures the fine-size reinforcement above the neat value (dotted), which the pure exponential (dashed) cannot represent.

The decreasing-only exponential cannot, by construction, exceed the matrix value, and therefore fails to represent the PCL 6800 modulus reinforcement at the fine sizes (Section 3.6). Adding an additive Halpin–Tsai-type reinforcement term, Equation (1) becomes

(2) $$P=P_{m}\left[exp\left(-a\varphi^{q}\right)+\beta \varphi \right]{\left(t/t_{ref}\right)}^{-1/m}$$

where *βφ* is the low-content limit of the Halpin–Tsai factor [59,60]. Adding this term improved the PCL 6800 modulus description substantially (Figure 9), raising the coefficient of determination from *R*^2^ = 0.79 to 0.92 and lowering the Akaike information criterion (*AIC*, a model selection metric that rewards goodness of fit while penalizing the number of free parameters, so that a lower value indicates a better-justified model) by about 29. For PCL 6500, the same term gave only a marginal improvement (ΔAIC ≈ −2), with *β* = 0.55 ± 0.29 not significantly different from zero, so it was not retained, confirming that the reinforcement is specific to the fine hemp/PCL 6800 combination. Because only one filler level (20 wt%) was tested for PCL 6800, the damage coefficient *a* and the reinforcement coefficient *β* cannot be separated from a single composite point; they were made identifiable by assuming no filler-induced damage at the two finest sizes (*a* = 0 at 0.08 and 0.25 mm), so that the observed stiffening is attributed entirely to *β*, while *P_m_* and *m* remain essentially unchanged. Under this assumption *β* = 1.49 ± 0.27, and the back-calculated effective filler modulus (approximately 0.7 to 1.2 GPa) is far below the intrinsic stiffness of hemp fibers, which is typically tens of GPa [12,16]. This result is expected for milled and partially debonded particles.

### 3.8. Best-Performing Formulation and Limitations

Within the experimental matrix investigated, PCL 6800 with 20 wt% hemp at 0.25 mm is the best-performing formulation. It combines the higher ductility and filler tolerance of the matrix with the fine-size plateau in strength and modulus. The 0.08 mm fraction gave statistically equivalent strength but is more demanding to mill and disperse. The most suitable particle size is, however, route-dependent. In solvent casting, the particle size directly interacts with the thickness of the film that is prepared. For injection molding, the 0.25 mm fraction represents a favorable compromise between mechanical performance and ease of powder handling, whereas fused deposition modeling would likely require the finer 0.08 mm fraction to satisfy the nozzle constraint, which is mechanically equivalent in strength. For the intended cosmetic packaging products, the final selection must also account for the appearance and surface texture conferred by the filler, which are not captured by the tensile response alone. The 20 wt% content is a compromise between a meaningful replacement of polymer by a natural filler and the strength penalty incurred at 30 wt%.

**Figure A4:**
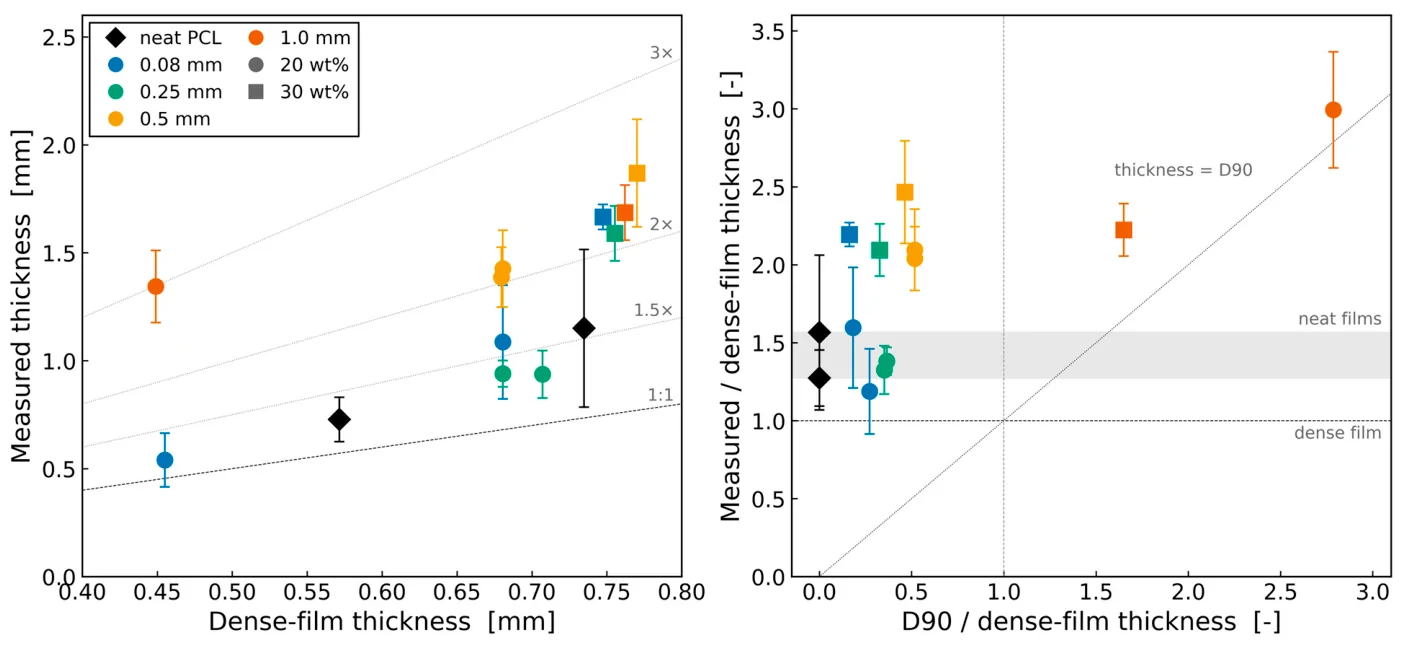
Thickness of the solvent-cast films: (**left**) measured specimen thickness (mean ± SD) versus the thickness of a dense, uniform film calculated from the poured masses, with the 1:1 line and the 1.5×, 2×, and 3× lines; (**right**) ratio of the two versus the D90 of the hemp fraction normalized to the dense-film thickness, with the dense-film (dashed) and thickness = D90 (dotted) lines and the range of the neat films (shaded). Color: particle size; marker: hemp content. The specimens were cut for tensile testing and are not necessarily representative of the whole film.

Several limitations should be acknowledged, framed by the scope of the study, which was conceived as a feasibility screening of solvent-cast PCL/hemp formulations preliminary to an ongoing melt processing investigation. Accordingly, the solvent casting results are used to pre-select the matrix grade, the filler content and the particle size window to be carried into each melt processing route, rather than as final property values. The molecular weight of the cast films was not measured, and the residual solvent content was measured only after storage (Section 3.3). Both are expected to affect mainly the absolute properties, since all the formulations of a grade shared the same dissolution and drying conditions and the residual solvent, controlled by diffusion, scales with film thickness, which is accounted for in the analysis; the two grades, however, may be affected to a different extent. Each formulation was cast once, and only two filler contents were tested, so the damage and reinforcement terms of Equation (2) cannot be separated for PCL 6800 without assumptions. The stresses were calculated with the measured specimen thickness, which accounts for the variability within the films but does not correspond to a dense cross-section, especially for the coarse fractions and at 30 wt% (Figure A4). Simply rescaling the load-bearing area would not capture this effect, since the stress intensification around voids and protruding particles plays a decisive role; a more refined description of how the solution fills the volume around fine and coarse particles would also be needed. XRD, molecular weight measurements, and fracture surface microscopy are planned for the melt processing phase.

The microstructure clarifies which limitations are specific to the processing route. The matrix bubbles and voids originate from air entrapment and from vapor evolution under the drying skin, both tied to the presence of a volatile solvent and a drying front [43], and are therefore expected to be largely absent in solvent-free melt processing. The features intrinsic to the filler, namely the intra-particle porosity of hemp, the weak filler–matrix interface, and the loss of dispersion quality with particle size, will persist regardless of the route. The relative ranking of formulations established here is therefore expected to carry over, whereas the absolute properties, depressed by the casting-specific defects, are not transferable and are used only for pre-selection. Reprocessing is also relevant to the planned melt processing routes: for biodegradable matrices filled with lignocellulosic fibers, the tensile properties were preserved for about three injection or extrusion cycles and then decreased, mainly because of fiber shortening and matrix degradation [61,62], while the filler dispersion improved [63]. The low aspect ratio of the milled hemp and the low processing temperature of PCL should mitigate both effects, which will be verified in the melt processing phase. The volume fractions were calculated from the skeletal density of the hemp and are therefore a lower bound (Section 2.1), so the back-calculated effective filler modulus is indicative. The experimental design is intentionally asymmetric, with PCL 6800 not tested at 30 wt% because of the higher solution viscosity, and the grade comparison is restricted accordingly. Within these boundaries, the solvent-cast results are used for the relative ranking of formulations rather than for absolute property determination, which is the appropriate role of a feasibility study.

## 4. Conclusions

Biodegradable PCL/hemp biocomposites were prepared by solvent casting and characterized by tensile testing, with the dataset analyzed statistically and described by a combined filler-plus-defect model. The main findings are as follows:

- Particle size strongly affected the tensile properties (*p* < 0.0001), with a plateau between 0.08 and 0.25 mm (no significant difference, *p_adj_* > 0.25) and a marked drop from 0.25 to 0.5 mm.
- Increasing the hemp content from 20 to 30 wt% reduced strength by a factor of 2.5 to 4 (*p* < 0.001), largely independent of thickness. This confirms that hemp acts predominantly as a filler.
- PCL 6500 was stiffer and stronger and PCL 6800 more ductile; the grade ranking at 20 wt% was particle size-dependent, with PCL 6800 giving higher strength at 0.25 and 0.5 mm.
- A combined model was fitted to the thickness-corrected data. It described the PCL 6500 strength with *R*^2^ = 0.979; the low Weibull modulus (*m* ≈ 3) supports a defect-controlled behavior associated with the solvent-cast route.
- For PCL 6800 at 20 wt%, the modulus at 0.08 and 0.25 mm exceeded that of the neat polymer (*p* = 0.045 and *p* = 0.003). This mild reinforcement, specific to the fine hemp/PCL 6800 combination, is captured by an additive Halpin–Tsai term.
- Cross-sectional microscopy and the tensile results indicated two origins of the reduced properties: process-related defects (matrix voids and bubbles left by solvent evaporation) and intrinsic features of the system (a weak filler/matrix interface and the porous structure of the hemp particles).
- DSC and TGA of the neat matrices showed that casting raised the crystallization temperature by 9 to 15 °C, consistent with a partial degradation during dissolution, without reducing the thermal stability, so the degradation remained limited and affects the absolute properties rather than the comparison between formulations.

Within the investigated space, PCL 6800 with 20 wt% hemp at 0.25 mm is the best-performing formulation. Solvent casting proved useful for the rapid screening of formulations. This feasibility study establishes the relationships between matrix grade, filler content, granulometry and tensile properties that will guide the next phase, in which the selected formulations will be processed by injection molding and fused deposition modeling (FDM). The two classes of defect are expected to behave differently in the transition: the process-related defects are tied to the presence of the solvent and drying kinetics and should be largely removed by melt processing. The absolute properties are then expected to improve. The intrinsic filler/matrix limitations will persist and, together with the size, content and grade ranking established here, are expected to carry over. In those routes the usable particle size is constrained by the process, finer for FDM to avoid nozzle clogging and coarser for injection molding, and the property–size relationship reported here allows for the finest processable fraction to be selected for each route, while the cosmetics packaging application adds appearance and surface texture as further selection criteria. Future work will also include XRD, molecular weight measurements, and fracture surface microscopy.

## Figures and Tables

**Table 1:** Investigated formulations.

Sample ID	PCL Grade	Hemp Content [wt%]	Particle Size [mm]
PCL6500	6500	0	-
PCL6500_20_1	6500	20	1.0
PCL6500_20_0.5	6500	20	0.5
PCL6500_20_0.25	6500	20	0.25
PCL6500_20_0.08	6500	20	0.08
PCL6500_30_1	6500	30	1.0
PCL6500_30_0.5	6500	30	0.5
PCL6500_30_0.25	6500	30	0.25
PCL6500_30_0.08	6500	30	0.08
PCL6800	6800	0	-
PCL6800_20_0.5	6800	20	0.5
PCL6800_20_0.25	6800	20	0.25
PCL6800_20_0.08	6800	20	0.08

**Table 2:** Characteristic diameters of milled hemp fractions from laser diffraction. Span is evaluated as (D90 − D10)/D50.

Milling	Nominal Sieve [mm]	D10 [μm]	D50 [μm]	D90 [μm]	Span [-]
Centrifugal	0.08	14.9	46.3	124.0	2.35
Centrifugal	0.25	21.3	78.8	248.6	2.89
Centrifugal	0.5	64.8	235.4	351.7	1.22
Centrifugal	1	302.5	809.2	1250.8	1.17
Knives	0.5	72.1	295.4	383.9	1.06
Knives	1	54.8	526.8	882.0	1.57

**Table 3:** DSC parameters of the as-received pellets and of the neat solvent-cast films (10 °C/min, one sample per material). Peak temperatures; *T_g_* at the midpoint of the step; *X_c_* = Δ*H_m_*/Δ*H_m_*^0^ with Δ*H_m_*^0^ = 148 J/g.

PCL Grade	Form	First Heating		Cooling		Second Heating			
		*T_m_* [°C]	*X_c_* [%]	*T_c_* [°C]	Δ*H_c_* [J/g]	*T_g_* [°C]	Δ*c_p_* [J/(g·K)]	*T_m_* [°C]	*X_c_* [%]
6500	pellet	65.1	44.4	14.0	57.4	−63.8	0.095	56.7	41.2
	film	64.1	52.2	28.9	58.5	−63.0	0.077	57.5	44.0
6800	pellet	62.4	45.5	18.2	54.5	−63.1	0.101	57.5	38.8
	film	67.2	52.7	27.1	58.0	−63.8	0.083	57.9	42.5

## Data Availability

The raw data supporting the conclusions of this article will be made available by the authors upon request.
